# Nix restores mitophagy and mitochondrial function to protect against PINK1/Parkin-related Parkinson’s disease

**DOI:** 10.1038/srep44373

**Published:** 2017-03-10

**Authors:** Brianada Koentjoro, Jin-Sung Park, Carolyn M. Sue

**Affiliations:** 1 Department of Neurogenetics, Kolling Institute of Medical Research, Royal North Shore Hospital, St. Leonards, New South Wales 2065, Australia; 2 Sydney Medical School-Northern, University of Sydney, St. Leonards, New South Wales 2065, Australia

## Abstract

Therapeutic targets are needed to develop neuroprotective treatments for Parkinson’s disease (PD). Mitophagy, the selective autophagic elimination of dysfunctional mitochondria, is essential for the maintenance of mitochondrial integrity and is predominantly regulated by the PINK1/Parkin-mediated pathway. Loss of function mutations in *Parkin* and *PINK1* cause an accumulation of dysfunctional mitochondria, leading to nigral neurodegeneration and early-onset PD with a high penetrance rate. We previously identified an asymptomatic homozygous *Parkin* mutation carrier who had not developed PD by her eighth decade despite the loss of functional Parkin. Here we discover a putative mechanism that protects her against PD. In contrast to Parkin-related PD patient-derived cells, the asymptomatic carrier cells show preserved mitochondrial function and mitophagy which is mediated by mitochondrial receptor Nip3-like protein X (Nix). Nix-mediated mitophagy was not affected by PINK1 knockdown. Both genetic and pharmacological induction of Nix restores mitophagy in PINK1- and Parkin-related PD patient cell lines, confirming its ability to induce mitophagy in the absence of PINK1/Parkin-mediated pathway. Moreover, Nix over-expression improves mitochondrial ATP production in these patient cells. Our results demonstrate that Nix can serve as an alternative mediator of mitophagy to maintain mitochondrial turnover, identifying Nix as a promising target for neuroprotective treatment in PINK1/Parkin-related PD.

Mitochondrial dysfunction plays a key role in the pathogenesis of Parkinson’s disease (PD)^1^,^2^,^3^,^4^,^5^. A fundamental process critical for maintaining healthy mitochondrial function is mitophagy, that is, the selective degradation of damaged mitochondria by autophagy^6^,^7^,^8^. Growing evidence suggests that dysregulation of mitophagy is implicated in the neurodegenerative process in PD^9^,^10^,^11^,^12^.

In mammals, mitophagy occurs during physiological processes such as reticulocyte maturation, which is mediated by the mitochondrial autophagy receptor Nip3-like protein X (Nix, also known as BCL2/adenovirus E1B 19 kDa interacting protein 3-like [BNIP3L])^13^,^14^. In addition, mitochondrial kinase PINK1 and cytosolic E3 ubiquitin ligase Parkin mediate mitophagy in response to mitochondrial damage^15^,^16^. During PINK1/Parkin-mediated mitophagy, Parkin is recruited to mitochondria in a PINK1 dependent manner upon the dissipation of mitochondrial membrane potential^17^,^18^,^19^ and thereby promotes ubiquitination and degradation of the outer mitochondrial membrane (OMM) proteins such as mitochondrial fusion proteins Mitofusin (Mfn) 1 and 2^20^,^21^,^22^. This process prevents the fusion of dysfunctional mitochondria with the healthy mitochondrial network and facilitates mitochondrial clearance via the autophagy-lysosomal system.

Loss-of-function mutations in *Parkin* (MIM# 602544)^23^ and *PINK1* (MIM# 608309)^24^ have been strongly associated with autosomal recessive early-onset PD. Autopsies of *Parkin* and *PINK1* mutation carriers have revealed dopaminergic neurodegeneration in the substantia nigra pars compacta^25^,^26^. Although bi-allelic mutations in *Parkin* typically have a high penetrance rate^27^,^28^, we have previously identified an unusual case of a homozygous *Parkin* mutation carrier who has not developed PD by her eighth decade despite Parkin deficiency (“asymptomatic carrier” hereafter), whereas her daughter who was a compound heterozygote presented with typical early-onset PD (“Parkin MT1” hereafter)^29^.

In this study, we identify Nix as the protective molecule which has prevented the asymptomatic carrier from developing PD. Using human fibroblasts as a model system, we confirm that Nix is responsible for facilitating mitochondrial elimination and thus supports normal mitochondrial function in the asymptomatic carrier, despite lack of the PINK1/Parkin pathway due to a demonstrated functional loss of Parkin. Moreover, we show that Nix restores mitophagy and improves mitochondrial function in fibroblasts derived from other Parkin- and PINK1-related early-onset PD patients, highlighting the broader therapeutic potential of Nix in PD.

## Results

### Mitochondrial function is normal in fibroblasts derived from the asymptomatic carrier despite the lack of functional Parkin

**Figure 1:**
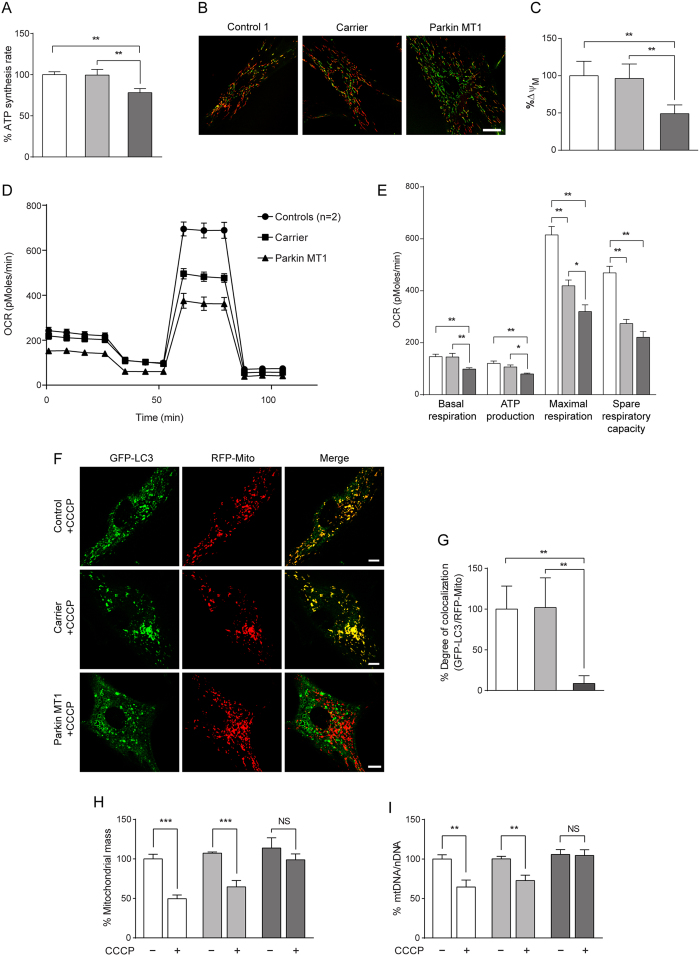
Mitochondrial function and mitophagy are preserved in fibroblasts derived from the asymptomatic carrier. Mitochondrial function was assessed in fibroblasts derived from healthy individuals (controls, n = 2; white bars), the asymptomatic carrier (light grey bars) and an affected Parkin-related PD patient (Parkin MT1; dark grey bars). As opposed to profound impairment in Parkin MT1, mitochondrial function in the asymptomatic carrier cells was preserved as shown by (**A**) the maximal rate of mitochondrial ATP synthesis, (**B**) representative images of cells stained with JC-1 (Scale bar: 25 μm), (**C**) mitochondrial membrane potential (Δψ_M_) determined by the ratio of red and green fluorescence signals of JC-1, (**D**) mitochondrial respiration determined by measuring oxygen consumption rate (OCR; mean ± SE) and (**E**) comparison of different aspects of mitochondrial respiration (mean ± SE). (**F**) Live cell imaging and (**G**) quantification of co-localization of autophagosomes and mitochondria were performed in the fibroblasts expressing GFP-LC3 (an autophagosomal marker, green signals in the left panels) and RFP-Mito (a mitochondrial marker, red signals in the middle panels). Exposure to CCCP caused a marked increase in co-localization of GFP-LC3 and RFP-Mito (yellow puncta in the right panels) in the control and asymptomatic carrier fibroblasts, while Parkin MT1 fibroblasts showed minimal overlapping. Scale bar: 10 μm. Elimination of mitochondria through CCCP-induced mitophagy was determined by measuring (**H**) mitochondrial mass using citrate synthase activity and (**I**) mitochondrial DNA (mtDNA) content relative to nuclear DNA (nDNA). Exposure to CCCP induced significant reduction of mitochondrial mass and mtDNA content in the controls and asymptomatic carrier fibroblasts, while no change was observed in the Parkin MT1 fibroblasts. NS; not significant, *p < 0.05, **p < 0.01, ***p < 0.001 in one-way ANOVA followed by *post hoc* Tukey’s HSD multiple comparison test (**A**,**C**,**E**,**G**) or in two-tailed Student’s *t*-test (**H**,**I**).

Mitochondrial dysfunction unequivocally contributes to Parkin- and PINK1-related PD^30^,^31^,^32^. However, we have reported unusual phenotypic variability in a single family despite the same molecular condition of Parkin deficiency^29^. To determine whether mitochondrial function was mechanistically linked to the differential phenotypic expression of Parkin deficiency, we measured mitochondrial energy production, membrane potential (Δψ_M_), oxygen consumption rate (OCR), and vulnerability to a mitochondrial complex I inhibitor rotenone^33^ in patient-derived cell lines from both the asymptomatic carrier and Parkin MT1 who is an affected family member of the asymptomatic carrier. Mitochondrial ATP synthesis rate (Fig. 1A) and Δψ_M_ measured using JC-1 (Fig. 1B,C) were significantly decreased in Parkin-MT1cells compared to the control cells, while the levels in asymptomatic carrier cells were unaffected. The asymptomatic carrier cells also depicted similar levels of OCR to the control cells under basal conditions and during ATP production, which were significantly higher than Parkin-MT1cells (Fig. 1D,E). Although maximal respiration was reduced in the asymptomatic carrier cells, the level was significantly higher than Parkin MT1 cells. There was no difference in spare respiratory capacity between the asymptomatic carrier cells and Parkin-MT1cells. In addition, the asymptomatic carrier cells displayed a similar degree of resistance to rotenone-induced cytotoxicity as the control cells (Supplementary Fig. S1).

These results indicate that mitochondrial function in the asymptomatic carrier cells, unlike the Parkin MT1 cells which have the same condition of Parkin deficiency, is mostly preserved.

### Exposure to CCCP induces Parkin-independent mitophagy in the asymptomatic carrier cells

Given the role of Parkin in the PINK1/Parkin-mediated mitophagy^16^, we next assessed the degradation of mitochondria using a mitochondrial protonophore carbonyl cyanide *m*-chlorophenyl hydrazone (CCCP) which is commonly used to induce mitochondrial fragmentation and mitophagy^17^,^18^,^19^. To detect mitophagy, we examined an autophagosomal engulfment of mitochondria using co-localization of Green Fluorescent Protein-tagged microtubule-associated protein light chain 3 (GFP-LC3)-labelled autophagosomes and mitochondria-targeted Red Fluorescent Protein (RFP-Mito)-labelled mitochondria by confocal microscopy (Fig. 1F,G), and changes in the mitochondrial mass by activity of a mitochondrial matrix protein citrate synthase^20^ (Fig. 1H) as well as mitochondrial DNA (mtDNA) content (Fig. 1I). Combination of these methods reliably detected the occurrence of CCCP-induced mitophagy in the control cells and the lack thereof in the Parkin MT1 cells, consistent with the previous study^34^, while no aberrant increase in co-localization between GFP-LC3 and RFP-Mito above basal levels was observed without CCCP treatment (Supplementary Fig. S2). Upon CCCP treatment, the asymptomatic carrier cells displayed a similar increase of co-localization between GFP-LC3 and RFP-Mito to the control cells (Fig. 1G), and a significant reduction of mitochondrial mass (Fig. 1H) and mtDNA content (Fig. 1I), indicating an induction of Parkin-independent mitophagy in the asymptomatic carrier cells. Notably, CCCP-mediated conversion of LC3-I to LC3-II^35^ occurred at a similar level in all cell lines studied (Supplementary Fig. S3), excluding non-specific degradation of mitochondria by hyperactive autophagy as the cause of mitophagy in the asymptomatic carrier cells.

### Parkin-independent mitophagy in the asymptomatic carrier cells is not dependent on PINK1

**Figure 2:**
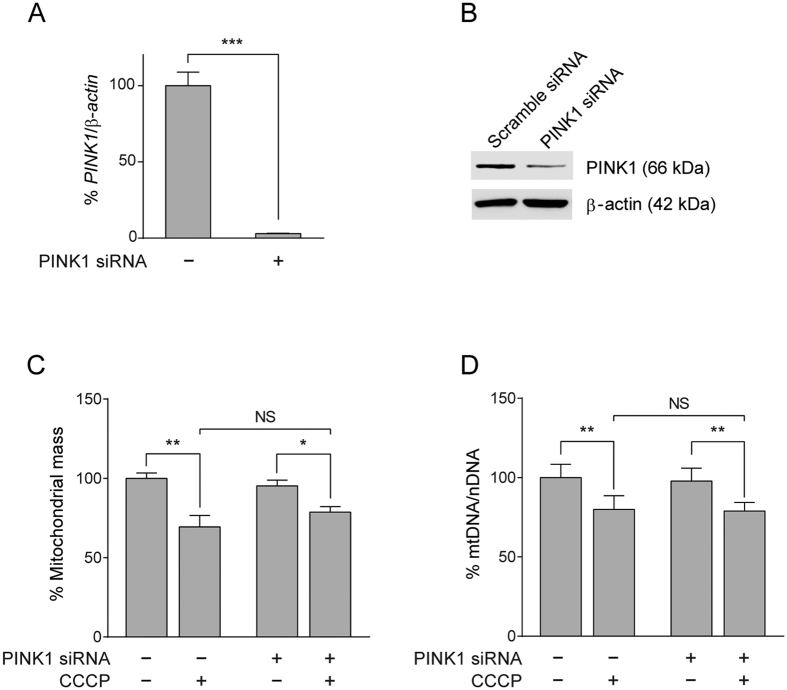
PINK1 is dispensable in the Parkin-independent mitophagy observed in fibroblasts derived from the asymptomatic carrier. Successful knockdown of PINK1 by siRNA in the asymptomatic carrier fibroblasts (light grey bars) resulted in >95% decrease of (**A**) *PINK1* transcripts (48 hours post transfection) followed by (**B**) a marked reduction of PINK1 protein (72 hours post transfection) compared to the scramble siRNA counterpart. Knockdown of PINK1 did not affect mitophagy in the asymptomatic carrier cells as demonstrated by the significant reduction of (**C**) mitochondrial mass and (**D**) mtDNA content by CCCP. NS; not significant, *p < 0.05, **p < 0.01, ***p < 0.001 in one-way ANOVA followed by *post hoc* Tukey’s HSD multiple comparison test (**A**,**C**,**D**) or in two-tailed Student’s *t*-test (**B**).

PINK1 has recently been demonstrated to recruit autophagy receptors independently of Parkin to promote mitophagy in HeLa cells^36^. To examine the possible involvement of PINK1 in the Parkin-independent mitophagy observed in the asymptomatic carrier cells, we first measured its expression levels in our cells. The asymptomatic carrier cells showed comparable levels of PINK1 to the controls regardless of CCCP treatment (Supplementary Fig. S4). Also, there was no increase of PINK1 detected in other Parkin patient cells used in this study (Supplementary Fig. S4). Moreover, the asymptomatic carrier cells, even after PINK1 was silenced using siRNA (Fig. 2A and B), consistently showed a significant reduction of mitochondrial mass (Fig. 2C) and mtDNA content (Fig. 2D) upon exposure to CCCP. These results indicate that PINK1 is dispensable in the Parkin-independent mitophagy observed in the asymptomatic carrier cells.

### Nix mediates Parkin-independent mitophagy in the asymptomatic carrier cells

**Figure 3:**
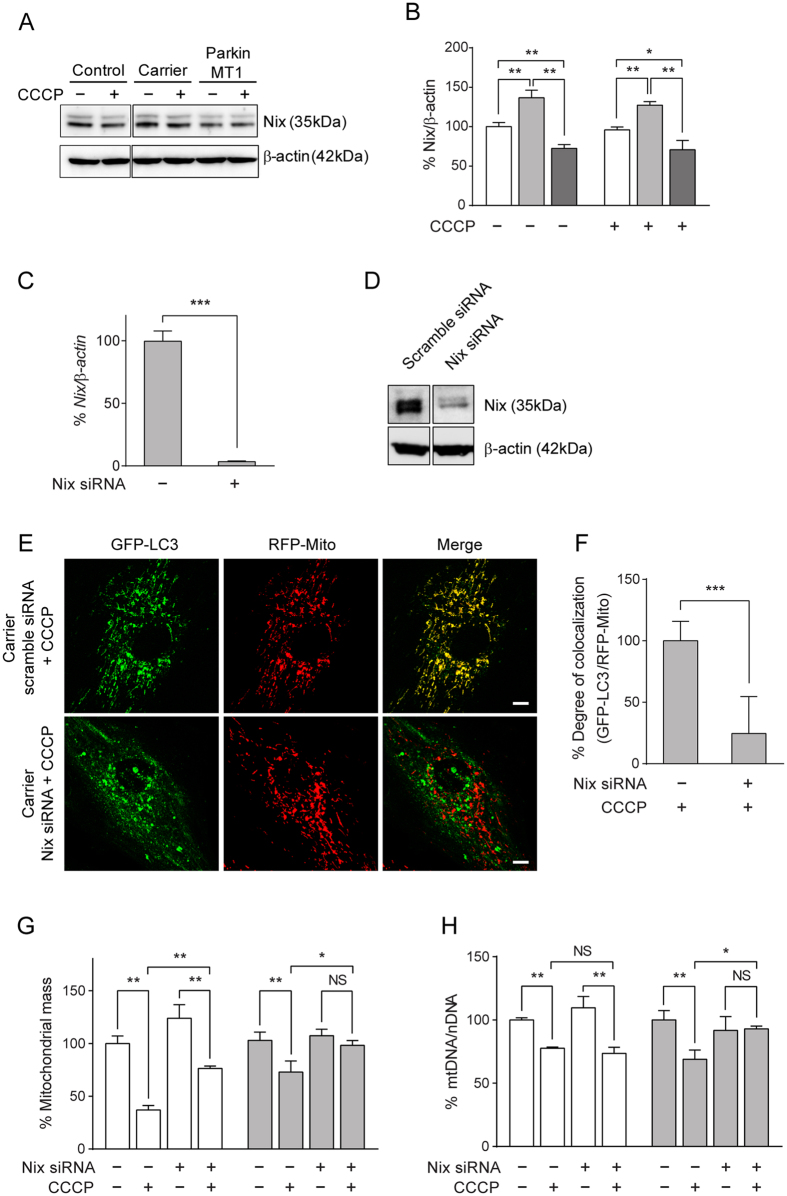
Identification of Nix as the mediator of the Parkin-independent mitophagy observed in fibroblasts derived from the asymptomatic carrier. (**A**) Immunoblotting of Nix (35 kDa) from cell lysates of the control (white bars), the asymptomatic carrier (light grey bars) and the Parkin MT1 (dark grey bars) revealed an upregulation of Nix in the asymptomatic carrier cells regardless of CCCP treatment. β-actin (42 kDa) was used as a loading control. (**B**) Densitometric analysis confirmed the visual observation of Nix expression levels. (**C**,**D**) Successful knockdown of *Nix* by siRNA in the asymptomatic carrier fibroblasts resulted in >95% decrease of (**C**) *Nix* transcripts (48 hours post transfection) followed by (**D**) a marked reduction of Nix protein (72 hours post transfection) compared to the scramble siRNA counterpart. (**E**) Knockdown of *Nix* abrogated CCCP-induced co-localization of GFP-LC3 and RFP-Mito in the asymptomatic carrier fibroblasts (bottom right panel), while the scramble siRNA counterpart (upper right panel) showed an increase in their co-localization indicative of mitophagy. Scale bar: 10 μm. (**F**) Degree of co-localisation was significantly decreased in the asymptomatic carrier fibroblasts transfected with Nix siRNA compared to the scramble siRNA counterpart. Nix siRNA, but not the scramble siRNA, blocked the CCCP-induced reduction in (**G**) mitochondrial mass and (**H**) mitochondrial DNA (mtDNA) content compared to nuclear DNA (nDNA) in the asymptomatic carrier fibroblasts (light grey bars), while either treatment did not affect CCCP-induced mitophagy in the control fibroblasts (white bars). NS; not significant, *p < 0.05, **p < 0.01, ***p < 0.001 in one-way ANOVA followed by *post hoc* Tukey’s HSD multiple comparison test (**B**,**G**,**H**) or in two-tailed Student’s *t*-test (**C**,**F**).

Given that Nix is a selective autophagic receptor located on the OMM and has previously been demonstrated to mediate mitochondrial clearance in primary mouse embryonic fibroblasts^37^, we next determined the expression levels of Nix in our Parkin patient cell lines. We found that the asymptomatic carrier cells expressed significantly higher levels of Nix compared to the control and Parkin MT1 cells at both protein (Fig. 3A,B) and mRNA levels (Supplementary Fig. S5). Notably, there was no compensatory increase of Nix detected in other Parkin patient cells used in this study (Supplementary Fig. S6). To test whether Nix played a role in the PINK1/Parkin-independent mitophagy observed in the asymptomatic carrier cells, we next silenced *Nix* using siRNA and assessed whether mitophagy was impaired. Successful knockdown of *Nix* in the asymptomatic carrier cells (Fig. 3C,D) caused a loss of CCCP-induced mitophagy as demonstrated by a marked reduction in co-localization of GFP-LC3 and RFP-Mito (Fig. 3E,F), and blocked the reduction of mitochondrial mass (Fig. 3G) and mtDNA content (Fig. 3H) compared to the scramble siRNA-transfected counterparts. These results indicate the direct involvement of Nix in the mitophagy observed in the asymptomatic carrier cells.

### Nix over-expression restores mitophagy and mitochondrial function in the Parkin- and PINK1-related PD patient cell lines

**Figure 4:**
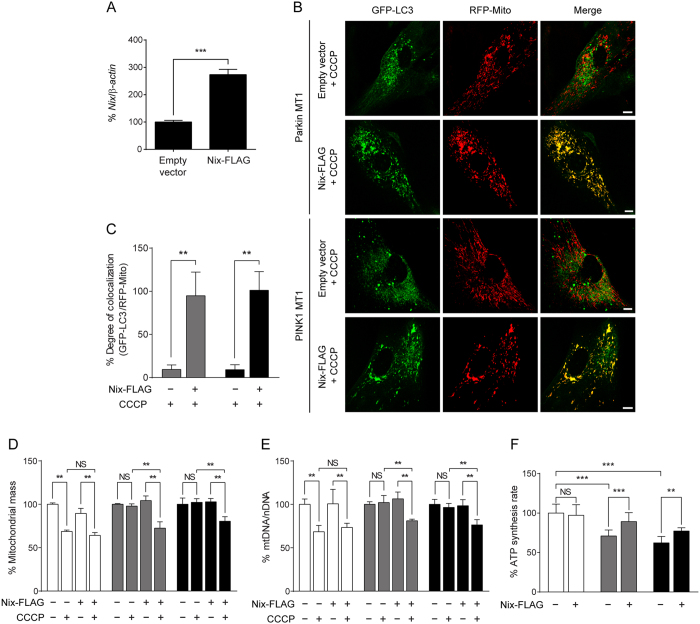
Nix over-expression restores mitophagy and improves mitochondrial function in fibroblasts derived from Parkin- or PINK1-related Parkinson’s disease patients. (**A**) Transduction of Nix-FLAG lentivirus in fibroblasts derived from a Parkin patient (Parkin MT1) resulted in about 3-fold increase of *Nix* expression (48 hours post transduction) compared to the empty vector counterpart. (**B**) Over-expression of Nix-FLAG increased co-localization of GFP-LC3 and RFP-Mito in fibroblasts derived from the Parkin MT1 and a PINK1 patient (PINK1 MT1) upon exposure to CCCP, while the empty vector transduced counterparts showed no change. (**C**) The effect of Nix-FLAG on the increased co-localization in the Parkin MT1 (dark grey bars) and PINK1 MT1 (black bars) was confirmed by calculating the degree of co-localization. Exposure to CCCP induced a significant reduction in (**D**) mitochondrial mass and (**E**) mitochondrial DNA (mtDNA) content compared to nuclear DNA (nDNA) in Nix-FLAG over-expressing fibroblasts derived from Parkin (Parkin MT1, MT2 and MT3; dark grey bars) and PINK1-related Parkinson’s disease patients (PINK1 MT1 and MT2; black bars), similar to those observed in the control fibroblasts (n = 3; white bars). (**F**) Parkin MT1 and MT2and PINK1 MT1patient fibroblasts expressing Nix-FLAG showed a significantly higher mitochondrial ATP synthesis rate compared to the empty vector expressing counterparts. NS; not significant, *p < 0.05, **p < 0.01, ***p < 0.001 in two-tailed Student’s *t*-test (**A**) or in one-way ANOVA followed by *post hoc* Tukey’s HSD multiple comparison test (**C**–**F**).

Our findings on the PINK1/Parkin-independent mitophagy in the asymptomatic carrier cells proposed that Nix might restore mitophagy and mitochondrial function in Parkin- and PINK1-related PD. To test this hypothesis, we over-expressed FLAG-tagged wild-type Nix using lentivirus (Fig. 4A) in fibroblast lines derived from three Parkin- (Parkin MT1, MT2 and MT3) and two PINK1- (PINK1 MT1 and MT2) related early-onset PD patients. These patient cells showed a loss of Parkin and/or CCCP-induced ubiquitination of Mfn2 (Supplementary Fig. S7). Notably, over-expressed Nix-FLAG mainly localized to mitochondria (Supplementary Fig. S8) and did not cause appreciable cell death (Supplementary Fig. S9A). Also, Nix over-expression, without CCCP treatment, did not increase mitophagy above basal levels as shown by lack of increase in co-localization of mitochondria and autophagosomes (Supplementary Fig. S10). When transduced with lentivirus carrying an empty vector, these patient cells were unable to induce mitophagy following exposure to CCCP (Fig. 4B–E), confirming a defective PINK1/Parkin mitophagy pathway in these cell lines. On the contrary, the patient cells over-expressing Nix showed a restoration of CCCP-induced mitophagy as demonstrated by a marked increase in the co-localization of GFP-LC3 and RFP-Mito (Fig. 4B,C) and a significant reduction of mitochondrial mass (Fig. 4D) and mtDNA content (Fig. 4E). Importantly, over-expression of Nix significantly increased the mitochondrial ATP synthesis rate in both the Parkin and PINK1 patient cells (Fig. 4F), indicating the functional improvement of mitochondria by restoring mitochondrial quality control through Nix-mediated mitophagy.

### Pharmacological induction of Nix promotes CCCP-induced mitophagy in the Parkin- and PINK1-related PD patient cell lines

**Figure 5:**
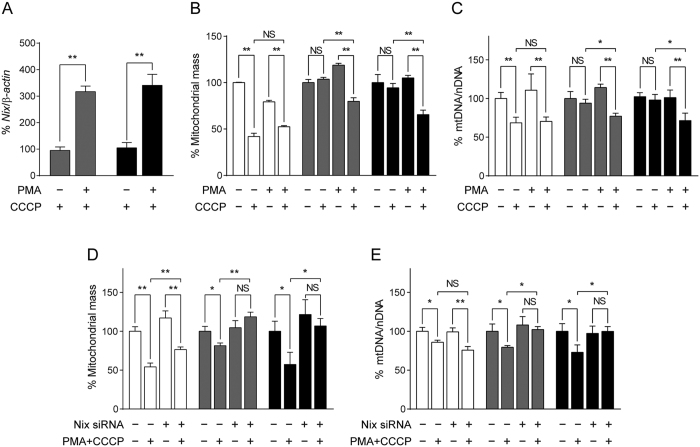
Pharmacological induction of Nix promotes CCCP-induced mitophagy in fibroblasts derived from Parkin or PINK1-related Parkinson’s disease patients. (**A**) Treatment of phorbol 12-myristate 13-acetate (PMA; 10 nM) significantly increased Nix expression in CCCP-treated fibroblasts derived from Parkin (Parkin MT1; dark grey bars) and PINK1 (PINK1 MT1; black bars) patients. PMA-treated Parkin MT1 and PINK1 MT1 fibroblasts showed a significant reduction in (**B**) mitochondrial mass and (**C**) mitochondrial DNA (mtDNA) content compared to nuclear DNA (nDNA) upon exposure to CCCP, indicative of mitophagy, while (**D** and **E**) knockdown of *Nix* using siRNA, but not the scramble siRNA, abolished the PMA-mediated restoration of CCCP-induced mitophagy in both patient fibroblasts. NS; not significant, *p < 0.05, **p < 0.01 in one-way ANOVA followed by *post hoc* Tukey’s HSD multiple comparison test (**A**–**E**).

Phorbol 12-myristate 13-acetate (PMA) is a protein kinase C activator which has been reported to induce Nix expression *in vitro*^38^. To test whether PMA could be used to promote mitophagy via induction of Nix expression, we treated our Parkin and PINK1 patient cells with PMA and assessed mitophagy. Upon induction of mitophagy by CCCP, we observed that PMA induced *Nix* expression in both Parkin MT1 and PINK1 MT1 cells (Fig. 5A), without detectable cytotoxicity (Supplementary Fig. S9B). Concurrently, degradation of mitochondria was observed in the PMA-treated Parkin and PINK1 patient cells as indicated by a significant reduction of mitochondrial mass (Fig. 5B) and mtDNA content (Fig. 5C). Furthermore, knockdown of *Nix* using Nix siRNA abolished the observed effect of PMA in restoring CCCP-induced mitophagy in these patient cells (Fig. 5D,E), confirming the involvement of Nix in this process.

## Discussion

We demonstrate that Nix provides an alternative mitophagy pathway that can restore mitochondrial function in the setting of PINK1 or Parkin deficiency. This highlights Nix as a potential therapeutic target for PD, given that mitochondrial dysfunction has been identified as a key contributing factor of dopaminergic neuronal vulnerability that is present in both familial and sporadic forms of PD^1^,^2^,^3^,^32^.

Impairment of mitochondrial function and mitophagy has previously been demonstrated in Parkin- and PINK1-mutant cell models^10^,^39^,^40^. In this study, we identified preserved mitochondrial function in the asymptomatic homozygous *Parkin* mutation carrier cells, despite the loss of functional Parkin^29^. Furthermore, successful induction of mitophagy in the asymptomatic carrier cells indicated the existence of an intact mitochondrial quality control system which was able to maintain normal mitochondrial function in the setting of Parkin deficiency.

Nix is an OMM-anchored autophagy receptor that mediates mitophagy in mammalian cells^13^,^14^,^37^. Here we discovered that Nix was up-regulated and mediated a Parkin-independent alternative mitophagy process in the asymptomatic carrier cells. The involvement of other types of Parkin-independent mitophagy is unlikely or, if any, minimal. A recent study in HeLa cells has demonstrated that PINK1 can recruit autophagy receptors, such as Nuclear domain 10 protein 52 (NDP52) and Optineurin (OPTN), to mitochondria to stimulate mitophagy directly, independently of Parkin^36^. However, we found that PINK1 was not a significant contributor to the Parkin-independent mitophagy in the asymptomatic carrier cells as mitophagy remained intact even after PINK1 knockdown. Moreover, Nix-mediated mitophagy is the predominant pathway in the asymptomatic carrier cells as evidenced by blockade of mitophagy following a knockdown of *Nix*. Collectively, these findings strongly support that Nix protects the asymptomatic carrier from developing PD by maintaining normal mitochondrial turnover and function.

Nix mediates mitophagy in erythrocytes under physiological conditions *in vivo* and Nix knockout mice exhibit a reduced number of mature erythrocytes, mitochondrial retention and defective removal of mitochondria by autophagosomes^13^. In addition, Nix can also be recruited by Parkin to rescue mitochondrial defects in pink1 mutant Drosophila^41^. Similarly, Nix has been shown to act as a substrate of Parkin in HEK293A cells and mediate mitophagy as part of the PINK1/Parkin pathway^41^. Intriguingly, the present study using fibroblasts derived from Parkin and PINK1-related PD patients provides unequivocal evidence that Nix can mediate mitophagy in the absence of Parkin or PINK1 activity. Importantly, restoration of mitochondrial turnover in these mutant cell lines through Nix over-expression improved mitochondrial function as indicated by the increase in the mitochondrial ATP production rate, highlighting the therapeutic potential of Nix to rescue mitochondrial dysfunction in PD via this mechanism. In addition, we also demonstrated the pharmacological induction of Nix-mediated mitophagy using PMA in the Parkin and PINK1 patient cells. PMA increases Nix transcription through the activation of PKCα and the binding of transcription factor Sp1 to GC-rich elements in the *Nix* promoter in cultured neonatal rat cardiac myocytes^38^. These findings support the potential use of pharmacological compounds to modulate Nix-mediated mitophagy as another therapeutic approach to PD. Given the role of mitochondrial dysfunction in neurodegeneration, drugs targeting Nix might have neuroprotective effects through the induction of this alternative mitophagy pathway to improve mitochondrial function and promote neuronal survival.

The mechanism by which Nix-mediated mitophagy is initiated in the asymptomatic carrier remains unknown. Given that phospho-ubiquitin is recognized as the key autophagy signal on mitochondria^42^,^43^, its role in the Nix-mediated mitophagy may be of interest. Moreover, it is also possible that a specific kinase may play a role in the activation of Nix-mediated mitophagy through phosphorylation of Nix or other intermediate autophagy molecules. A previous study has demonstrated that Nix is readily phosphorylated by Casein Kinase 2 at Ser117, Ser118 and Ser120 in HEK 293T cells^44^; however, the significance of these modifications to induce mitophagy is yet to be determined. Although further work to determine the precise molecular mechanisms that regulate Nix-mediated mitophagy is warranted, it may involve GTPase Ras homolog enriched in brain protein (Rheb), as their interaction has been demonstrated to promote mitophagy during high oxidative phosphorylation activity^45^. Melser *et al*. proposed that Rheb is recruited to the OMM to facilitate the interaction between Nix and LC3, thus promoting the engulfment of mitochondria by nascent autophagosomes^45^. Further studies are needed to determine whether Nix-mediated mitophagy can be induced to ameliorate the mitochondrial defects observed in human dopaminergic neurons and *in vivo* models of PD.

In conclusion, we identify Nix as an alternative mediator of mitophagy to maintain mitochondrial function, compensating for impaired PINK1/Parkin-mediated mitophagy in fibroblasts derived from the asymptomatic homozygous *Parkin* mutation carrier. Using genetic and pharmacological induction, we confirmed the ability of Nix to facilitate alternative mitophagy, which resulted in improvement of mitochondrial function in Parkin- and PINK1-related PD patient cells. Our study highlights Nix as a promising new therapeutic target for developing neuroprotective treatment of PD.

## Materials and Methods

### Cell culture

Establishment and culture of human fibroblasts and human olfactory neurosphere cell lines was performed as previously described^29^. Cells were subcultured to a maximum of 15 passages for all experiments. This study was approved by the Northern Sydney & Central Coast Health Human Research Ethics Committee.

### Lentivirus production and establishment of cell lines

The full-length *Nix* cDNA was amplified from pCMV6-Nix (Origene) in a PCR reaction using a forward primer containing an EcoRI site and a reverse primer containing a FLAG tag and a NotI site (Supplementary Table S1). After digestion with EcoRI and NotI, the PCR fragment was subcloned in the pER4 lentivirus vector, resulting in pER4-Nix-FLAG. The correct insertion of the fragment was confirmed by direct sequencing. Lentivirus for the expression of GFP-tagged LC3 (pLenti6-GFP-LC3, a generous gift of Dr Ernst Wolvetang, University of Queensland, Australia) and FLAG-tagged Nix (Nix-FLAG) was produced by transfecting each plasmid with the Lenti-X Lentiviral Packaging mix (Clontech) into 293 T cells using lipofection. The media containing lentivirus was collected at 48 and 72 hours post-transfection followed by concentration using a Lenti-X concentrator (Clontech) before determination of virus titre using a Lenti-X qRT-PCR titration kit (Clontech).

For generation of stable cell lines expressing GFP-LC3, fibroblasts were transduced with 1 multiplicity of infection (MOI) lentivirus in the presence of 4 μg/mL polybrene (Sigma) for 24 hours and subsequently grown in culture media containing 2 μg/mL blasticidin (Invitrogen) for selection. The established fibroblast lines were subsequently transduced with 10 MOI of lentivirus carrying either Nix-FLAG or empty vector.

### Assessment of mitochondrial ATP production rate

Mitochondrial ATP production rate was determined as previously described^46^. Briefly, fibroblasts were harvested by trypsinisation followed by determination of the total protein concentration using BCA protein assay kit (Thermo Scientific) according to the manufacturer’s instructions. Cells were diluted in a cell suspension buffer (150 mM KCl, 25 mM Tris-HCl pH 7.6, 2 mM EDTA pH 7.4, 10 mM KPO_4_ pH 7.4, 0.1 mM MgCl_2_ and 0.1% (w/v) BSA) at 1 mg/mL total protein. ATP synthesis was induced by incubation of 250 μL of the cell suspension with 750 μL of a substrate buffer (10 mM malate, 10 mM pyruvate, 1 mM ADP, 40 μg/mL digitonin and 0.15 mM adenosine pentaphosphate) for 10 minutes at 37 °C. Following this incubation, the reaction was stopped by adding 450 μL of a quenching buffer (100 mM Tris-HCl, 4 mM EDTA pH 7.75) to 50 μL aliquot of the reaction mixture and incubating for 2 minutes at 100 °C. The resulting reaction mixture was further diluted 1:10 in the quenching buffer and the amount of ATP was measured in an FB10 luminometer (Berthold Detection Systems) with an ATP Bioluminescence Assay Kit (Roche Diagnostics).

### Measurement of mitochondrial membrane potential

Mitochondrial membrane potential (Δψ_M_) was determined using JC-1 as previously described^46^. Briefly, fibroblasts were plated in a 35 mm μ-dish at 50,000 cells per dish and cultured for 24 hours. On the day of assay, the cells were incubated with 500 nM JC-1 (Cayman chemical). Fluorescence was visualized using a Leica SP5 confocal microscope (Leica) with constant parameters applied to acquire images from all samples. The area occupied by mitochondria in red fluorescence vs. mitochondria in green fluorescence per cell was calculated using Image J software (version 1.50i).

### Determination of oxygen consumption rate

Mitochondrial oxygen consumption rate (OCR) was determined by Seahorse Flux Analyzer (Agilent Technologies) as previously published^47^,^48^. Briefly, fibroblasts were plated at 60,000 per well in an XF24 V7 cell culture microplate and cultured overnight under standard culture conditions (37 °C, 5% CO2). After changing the media to pre-warmed basal media (XF Base media supplemented with 2 mM L-glutamine, pH 7.35 ± 0.05) and incubating at 37 °C for 1 hour in a non-CO2 incubator, the microplate was assembled with a hydrated sensor cartridge and placed in a XF24-3 extracellular flux analyzer. OCR was measured three times for 3 min after the stepwise injection of each compound; 1 μM oligomycin, 1 μM carbonyl cyanide-4-(trifluoromethoxy)phenylhydrazone (FCCP) and 0.5 μM Rotenone/antimycin A. Data was normalized to the number of cells per well using the CyQuant cell proliferation assay (Life Technologies). For comparison of OCR between control and patient cells, the third OCR measurement acquired after the addition of each compound was used to calculate (1) basal respiration (basal OCR – non-mitochondrial respiration), (2) ATP production (basal OCR – OCR measured after oligomycin injection), (3) maximal respiration (OCR measured after FCCP injection – OCR measured after oligomycin injection) and 4) spare respiratory capacity (maximal respiration – basal respiration).

### Cytotoxicity test

Cell death was determined using Thiazolyl Blue Tetrazolium Bromide (MTT; Sigma) and CytoTox 96 Non-Radioactive Cytotoxicity Assay Kits (Lactate Dehydrogenase, LDH; Promega) according to the manufacturer’s protocol. In brief, human olfactory neurosphere cells were seeded in 24-well culture plate at 50,000 cells per well and cultured for 48 hours. Cells were then exposed to increasing doses of rotenone (Sigma; 1.5 and 2.5 μM) for 72 hours.

For the MTT assay, cells were incubated in 0.2 M of MTT solution for 4 hours at 37 °C. The media was then removed and the formazan crystals were solubilised by addition of 0.04 M HCl (in isopropanol), followed by incubation at room temperature for 30 min with agitation. Absorbance of the MTT formazan at 570 nm and the background at 655 nm was measured using a Benchmark Microplate Reader (BioRad).

For the LDH assay, a 50 μL aliquot of culture media was incubated with a substrate mix for 30 minutes. Lactate dehydrogenase activity was measured spectrophotometrically at 490 nm.

### Assessment of mitophagy

To assess co-localisation of mitochondria and autophagosomes, 50,000 fibroblasts expressing GFP-LC3 were seeded on to a 35 mm μ-Dish (Ibidi), followed by transduction with CellLight Mitochondria-RFP BacMan 2.0 (Invitrogen) to label mitochondria according to the manufacturer’s instructions. Following 24 hours incubation, the cells were treated with either DMSO or 20 μM carbonyl cyanide 3-chloro phenylhydrazone (CCCP) (Sigma) for 2–4 hours to induce mitophagy and subjected to live cell imaging using a Leica TCS SP5 II confocal microscope (Leica Microsystems). Images of 50 individual cells from at least three independent experiments were taken and analysed. The degree of co-localisation was determined using a LAS-AF software v.2.6.0 (Leica Microsystems) as follows: degree of co-localisation [%] = area co-localisation ÷ area foreground with threshold and background subtraction set at 30%; area foreground = area image − area background.

For measurement of changes in the mitochondrial mass and mitochondrial DNA (mtDNA) content, fibroblasts were seeded in a 6-well culture plate at 200,000 cells per well and treated with either DMSO or 10 μM CCCP (Sigma) for 24 hours to induce mitophagy. Activity of citrate synthase (a mitochondrial matrix enzyme) was determined to assess changes in the mitochondrial mass using a Citrate Synthase Assay Kit (Sigma) according to the manufacturer’s instructions. Fibroblasts were harvested with a cell scraper, resuspended in a cell lysis buffer (CelLytic M Cell Lysis Reagent supplemented with a cocktail of protease inhibitors (Sigma)) and then briefly sonicated. After quantifying protein concentration, 10 μg of total protein was mixed with the provided substrate buffer followed by the addition of 100 μM oxaloacetate solution to start the reaction. Optical absorbance of the reaction mixture at 412 nm (OD_412_) was taken every 10 seconds for 1.5 minutes before and after the addition of the oxaloacetate solution. Citrate synthase activity was determined by subtracting the OD_412_ per minute before addition of oxaloacetate from OD_412_ per minute after addition of oxaloacetate.

Quantification of mtDNA was carried out using real time quantitative polymerase chain reaction (qPCR) as previously reported^49^. In brief, fibroblasts were harvested with a cell scraper. Total DNA was then extracted using a QIAamp DNA Mini Kit (Qiagen) according to the manufacturer’s instructions. A multiplex qPCR analysis was performed using TaqMan Gene Expression Master Mix (Invitrogen) on a Rotor Gene 6000 (Qiagen) according to the manufacturer’s instructions. The primers and TaqMan probes used in the reaction are listed in Supplementary Table S2. The amount of mtDNA was calculated relative to nuclear DNA (nDNA) using a Rotor-Gene 6000 Series Software v.1.7.

### Western blot

Protein expression levels were determined by Western blotting as follows. 20 to 30 microgram of total cell lysates was resolved using NuPAGE Novex 4–12% Bis-Tris SDS/polyacrylamide gels (Invitrogen) and transferred to a polyvinylidene fluoride membrane. The proteins blotted in the membrane were then probed with a sequential application of protein-specific primary antibodies and horseradish peroxidise–conjugated secondary antibodies (Supplementary Table S3). Chemiluminescence was developed using a SuperSignal West Pico or Femto Chemluminescent Substrate (Thermo Scientific) and detected using a LAS4000 (Fujifilm).

### Total RNA extraction and quantitative real time RT-PCR

Total RNA from fibroblasts was prepared using a RNeasy Mini Kit (Qiagen) and then reverse-transcribed into cDNA with a SuperScript III First-Strand Synthesis System (Invitrogen) following the manufacturer’s instructions. The resulting cDNA was used to determine gene expression in a quantitative real time RT-PCR (qRT-PCR) using a QuantiTect SYBR Green PCR Kit (Qiagen) on a Rotor Gene 6000 (Qiagen) according to the manufacturer’s instructions. The primers used in the reaction are listed in Supplementary Table S4.

### Small interfering RNA-mediated knockdown

Knockdown of *PINK1* and *Nix* in fibroblasts was achieved using Dharmacon ON-TARGET plus SMART pool-Human PINK1 (L-004030-00-0005, Thermo Scientific) and BNIP3L (L-011815-00-0005, Thermo Scientific) respectively and DharmaFECT1 siRNA Transfection Reagent (Thermo Scientific) following the manufacturer’s instructions. ON-TARGET plus Non-Targeting siRNA#1 (scramble siRNA; D-001810-01-05, Thermo Scientific) was used as a scramble control.

Gene knockdown was confirmed 48–72 hours post transfection using qRT-PCR and Western blotting. Greater than 95% reduction in the target mRNA level was regarded as successful knockdown.

### Statistical analysis

All results are representative of, at least, three independent experiments and expressed as mean ± standard deviation (SD), unless otherwise specified. Two-tailed Student’s *t*-test or one-way ANOVA followed by *post hoc* Tukey′s HSD test was used to test statistical significance. A *p* value of less than 0.05 was considered to be significant.

## Additional Information

**How to cite this article**: Koentjoro, B. *et al*. Nix restores mitophagy and mitochondrial function to protect against PINK1/Parkin-related Parkinson’s disease. *Sci. Rep.* **7**, 44373; doi: 10.1038/srep44373 (2017).

**Publisher's note:** Springer Nature remains neutral with regard to jurisdictional claims in published maps and institutional affiliations.

## Supplementary Material

Supplementary Figures and Tables
