# Supplementary material for: Nix restores mitophagy and mitochondrial function to protect against PINK1/Parkin-related Parkinson’s disease
Source: Sci Rep. 2017 Mar 10;7:44373. doi: 10.1038/srep44373 (PMC5345073; doi:10.1038/srep44373)
Supplement: Supplementary Figures and Tables [file srep44373-s1.pdf]

Nix restores mitophagy and mitochondrial function to protect against PINK1/Parkin-related Parkinson's disease

Brianada Koentjoro, PhD[1,2,*], Jin-Sung Park DVM, PhD[1,2,*], Carolyn M. Sue, MBBS, PhD[1,2,†]

[1]Department of Neurogenetics, Kolling Institute of Medical Research, Royal North Shore Hospital, St. Leonards, New South Wales 2065, Australia

[2]Sydney Medical School Northern, University of Sydney, St. Leonards, New South Wales 2065, Australia

[†]Corresponding author: Carolyn M. Sue

Department of Neurology, Royal North Shore Hospital and Kolling Institute of Medical Research, University of Sydney, St Leonards, NSW 2065, Australia. Tel: +61 2 9463 1828, Fax: +61 2 9463 1058. Email: carolyn.sue@sydney.edu.au

[*]These authors contributed equally to this work

Supplementary Materials

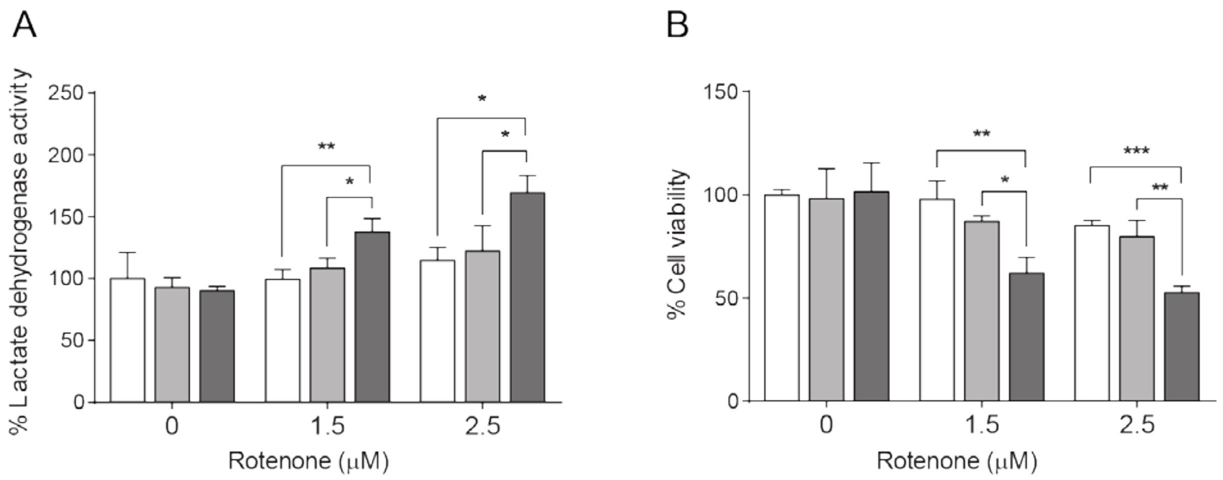

Supplementary Fig. S1. Asymptomatic carrier cells show resistance to rotenone induced cytotoxicity. Human olfactory neurosphere cells derived from healthy individuals (controls, n=3; white bars), the asymptomatic carrier (light grey bars) and the Parkin MT1 patient (dark grey bars) were tested for rotenone vulnerability with increasing doses of rotenone (0 - 2.5 μM for 72 hours) using (A) lactate dehydrogenase assay and (B) MTT assay. Asymptomatic carrier cells showed a similar degree of rotenone-induced cytotoxicity to the control. *; p<0.05, **; p<0.01, ***; p <0.001 in one-way ANOVA followed by *post hoc* Tukey's HSD multiple comparison test.

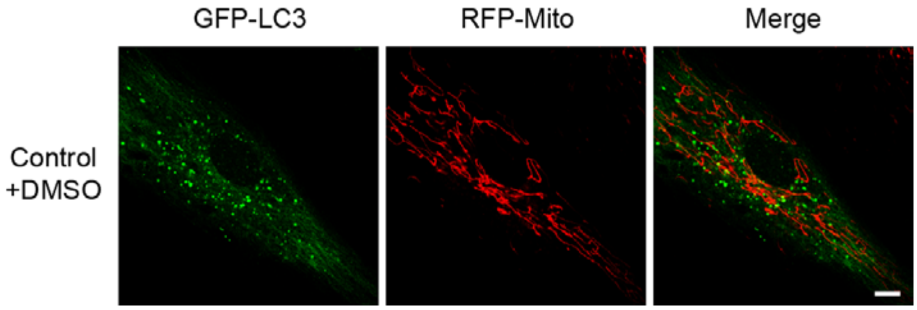

Supplementary Fig. S2. Absence of aberrant mitophagy by over-expression of GFP-LC3 and RFP-Mito in fibroblasts. Control fibroblasts expressing GFP-LC3 (an autophagosomal

marker, green signals in the left panel) and RFP-Mito (a mitochondrial marker, red signals in the middle panel) showed a minimal overlapping between the two markers (right panel) under basal conditions, indicating absence of abnormal mitophagic activity.

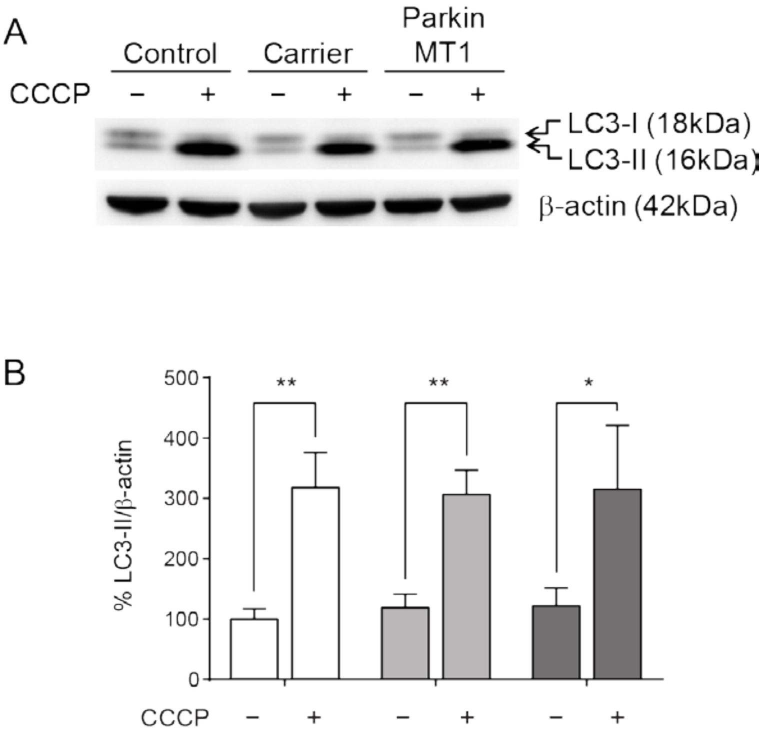

Supplementary Fig. S3. Similar levels of autophagy induced by CCCP treatment in fibroblasts. (A) Immunoblotting of LC3 showed a conversion of LC3-I (18 kDa) to LC3-II (16 kDa) in fibroblasts from a healthy control (white bars), the asymptomatic carrier (light grey bars) and the Parkin MT1 (dark grey bars) following 10 μM CCCP treatment for 6 hours. β-actin (42 kDa) was used as a loading control. (B) Densitometric analysis showed a similar ratio of LC3-II to β-actin in all cell lines tested here. *; $p<0.05$, **; $p<0.01$ in two-tailed Student's *t*-test.

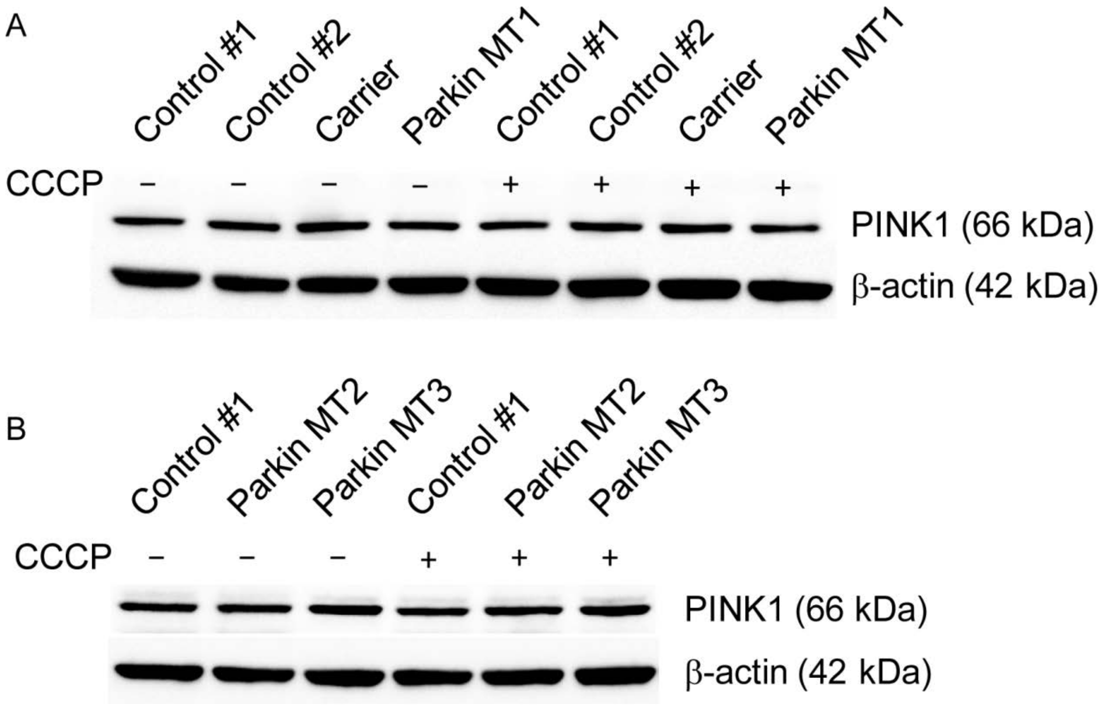

Supplementary Fig. S4. Expression of PINK1 in control and Parkin patient fibroblasts. Immunoblotting of PINK1 (66 kDa) from cell lysates of healthy controls (n=2) and in (A) the asymptomatic carrier cells, Parkin MT1, and (B) Parkin MT2 and MT3. β-actin (42 kDa) was used as a loading control.

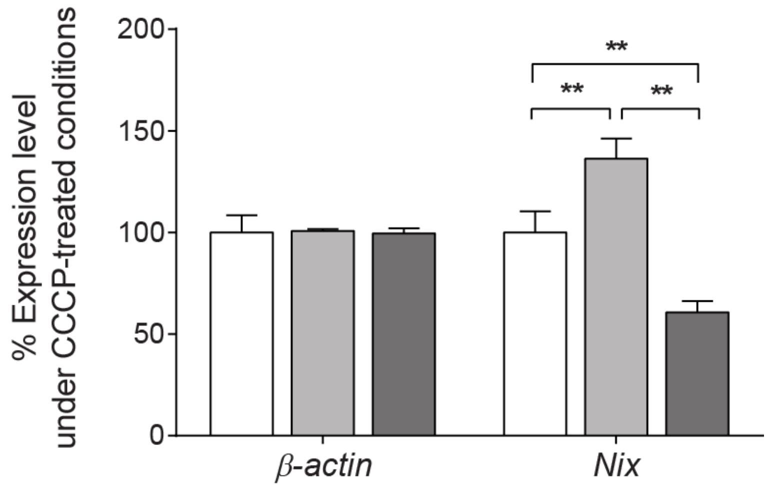

Supplementary Fig. S5. Up-regulation of *Nix* transcripts in CCCP-treated fibroblasts derived from the asymptomatic carrier. Expression of *Nix* was significantly elevated in the asymptomatic carrier (light grey bars) compared to the controls (n=2, white bars) and the Parkin MT1 (dark grey bars) fibroblasts in CCCP (10 μM for 6 hours) treatment conditions. *β-actin* was used as a housekeeping gene. **; p<0.01 in one-way ANOVA followed by *post hoc* Tukey's HSD multiple comparison test.

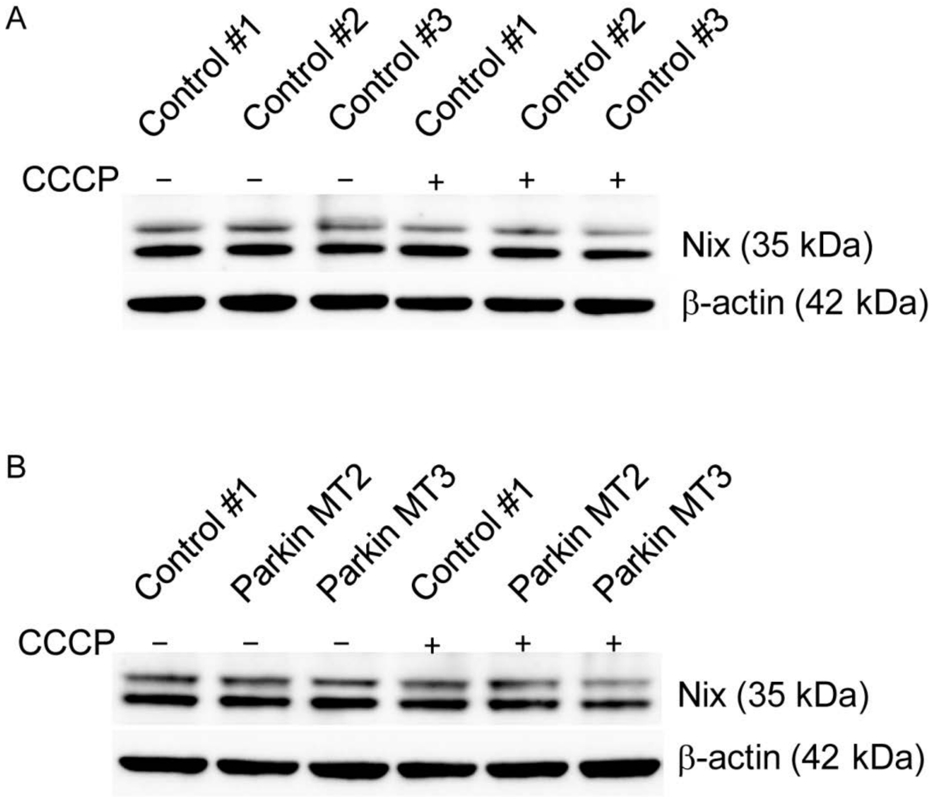

Supplementary Fig. S6. Expression of Nix in control and Parkin patient fibroblasts. Immunoblotting of Nix (35 kDa) from cell lysates of (A) healthy controls (n=3) and (B) Parkin MT2 and MT3. β-actin (42 kDa) was used as a loading control.

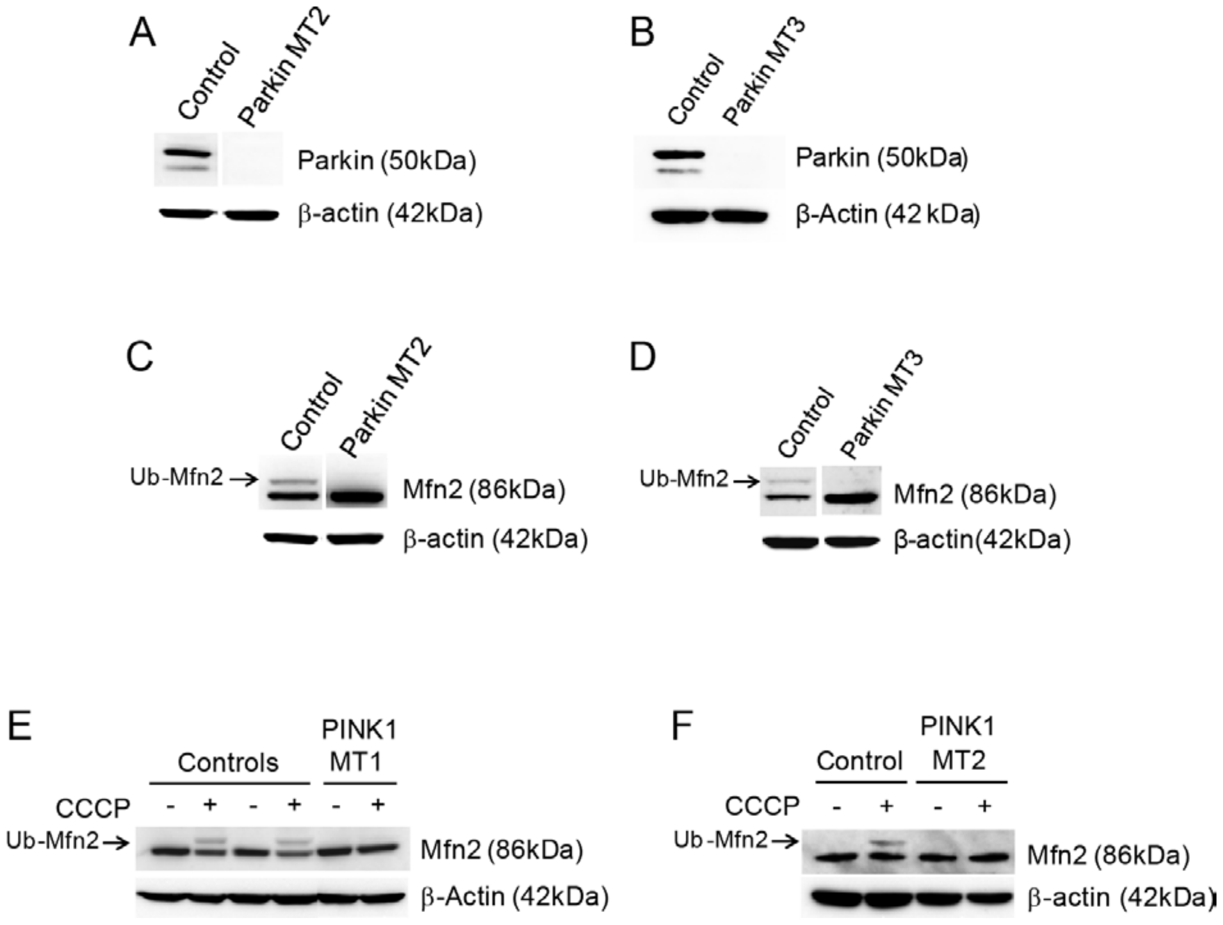

Supplementary Fig. S7. Loss of PINK1/Parkin-mediated ubiquitination of Mitofusin 2 (Mfn2) in fibroblasts derived from Parkin- and PINK1-related Parkinson's disease patients. (A, B) Immunoblotting revealed absence of wild-type Parkin (50 kDa) in Parkin MT2 and Parkin MT3 fibroblasts. (C-F) Ubiquitination of Mfn2 was induced by CCCP treatment (10 μM for 6 hours) in the controls as indicated by appearance of ubiquitinated Mfn2 (Ub-Mfn2) and decrease in native Mfn2, while (C) Parkin MT2, (D) Parkin MT3, (E) PINK1 MT1 and (F) PINK1 MT2 fibroblasts did not respond to the same treatment, indicating loss of the PINK1/Parkin-mediated ubiquitination of Mfn2. β-actin (42 kDa) was used as a loading control.

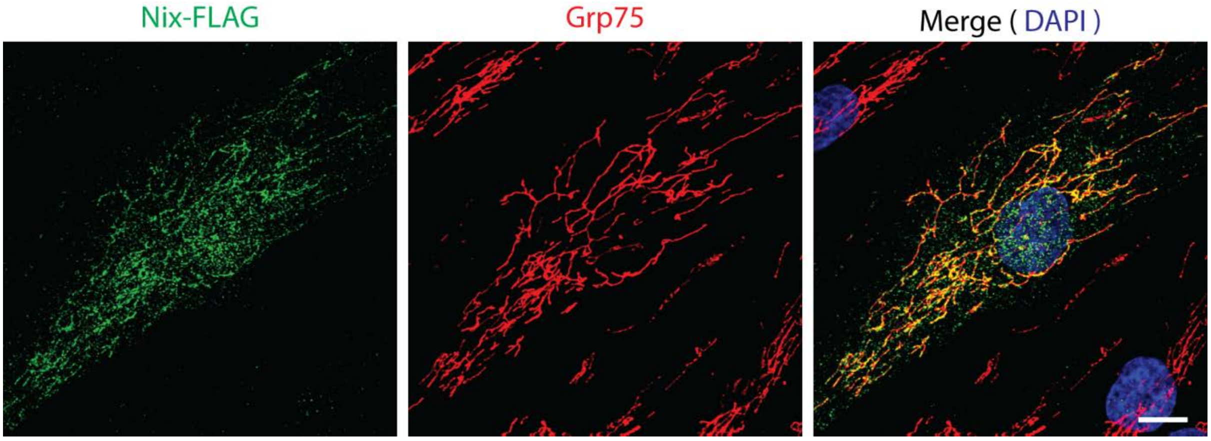

Supplementary Fig. S8. Localization of Nix-FLAG to mitochondria. Nix-FLAG (green) were mostly observed in mitochondria visualized by a mitochondrial marker Grp75 (red) while some signals were also detected in the cytosol and nucleus, indicating that Nix-FLAG is targeted to mitochondria. Scale bar: 10 µm.

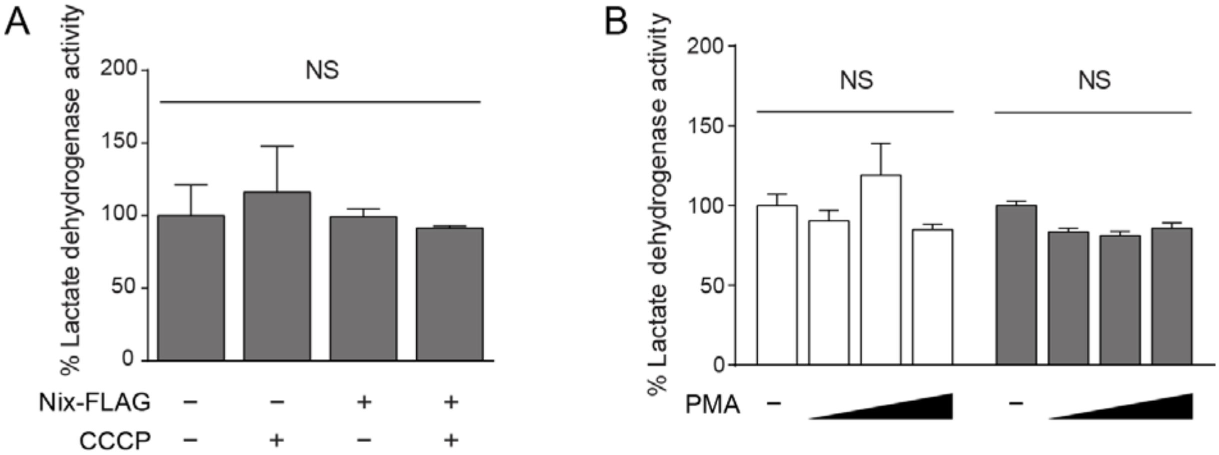

Supplementary Fig. S9. Lack of increased cell death by lentivirus transduction and phorbol 12-myristate 13-acetate (PMA) treatment in fibroblasts. Measurement of lactate dehydrogenase activity following (A) 72 hours after transduction of Nix-FLAG expressing lentivirus with or without CCCP treatment (10 µM for 24 hours) and (B) 24 hours exposure to increasing doses of PMA (0 - 50 nM) in fibroblasts derived from a healthy control (white bars) and a Parkin patient (Parkin MT1; dark grey bars). There was no increased cell death

detected in either treatment. NS; not significant in one-way ANOVA followed by *post hoc* Tukey's HSD multiple comparison test.

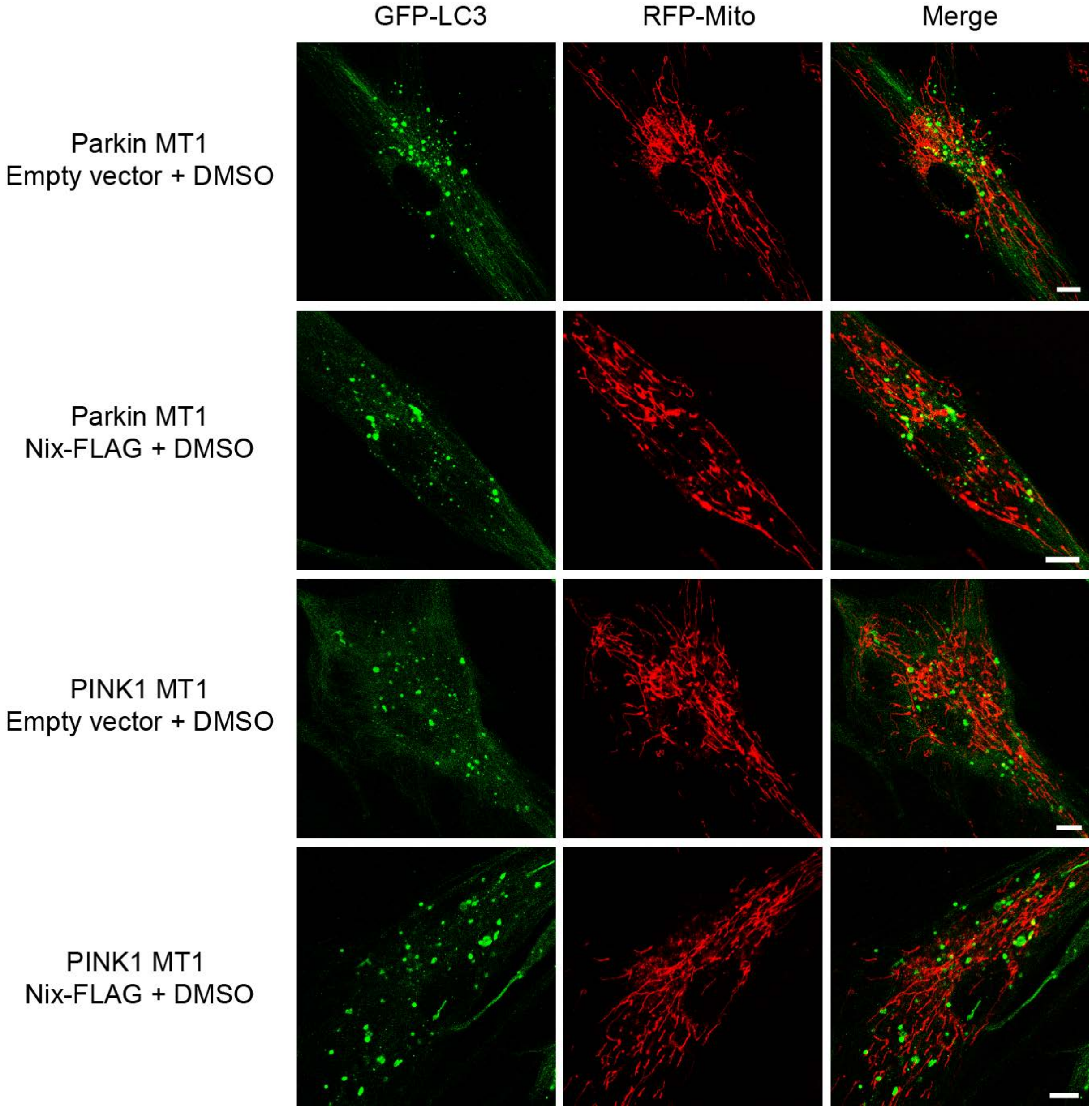

Supplementary Fig. S10. Lentivirus-mediated over-expression of Nix did not induce aberrant mitophagy. Nix over-expression alone did not visually increase co-localization of GFP-LC3 (green signals in the left panel) and RFP-Mito (red signals in the middle panel). Scale bar: 10 µm.

Supplementary Table 1. Sequence of primers for cloning

Primers	Restriction enzyme site & tag	Sequence (5′-3′)
Forward	EcoRI site	GGAATTC GTCGACTGGATCCGGTAC
Reverse	NotI site and FLAG tag	GGTCTAGAGCGGCCGCTTAAACCTTATCGTCGTCATCCTTGTAATCACGCGT GTAGGTGCTGGCAG

Supplementary Table 2. Sequence of primers and probes for quantification of mitochondrial DNA

Primers	Forward (5′-3′)	Reverse (5′-3′)
mtDNA	AGGACAAGAGAAATAAGGCC	TAAGAAGAGGAATTGAACCTCTGACTGTAA
nDNA	TTTTTGTGTGCTCTCCCAGGTCT	TGGTCACTGGTTGGTTGGC
**Probes**	**Sequence (5′-3′)**	
mtDNA	VIC-TTCACAAAGCGCCTTCCCCCGTAAATGA-TAMRA	
nDNA	FAM-CCCTGAACTGCAGATCACCAATGTGGTAG-TAMRA	

Supplementary Tab. 3. List of antibodies for detection of protein in Western blotting

Antibodies	Suppliers	Dilution	Condition
Parkin	Cell Signalling Technology, Inc., Denver, MA, USA	1:1000	1% skim milk 0.05% TBST, 4°C 16 hours
Mfn2	Abcam, Cambridge, MA, USA	1:2000	1% skim milk 0.05% TBST, 4°C 16 hours
LC3	Medical & Biological Laboratories Co., Ltd., Nagoya, Japan	1:1000	1% skim milk 0.05% TBST, 4°C 16 hours
Nix	Abcam, Cambridge, MA, USA	1:1000	5% skim milk 0.05% TBST, 4°C 16 hours
PINK1	Abcam, Cambridge, MA, USA	1:500	5% skim milk 0.05% TBST, 4°C 16 hours
β-actin	Sigma, St. Louis, MO, USA	1:5000	5% skim milk 0.05% TBST, 4°C 16 hours
Anti-mouse IgG	Bio-Rad, Hercules, CA, USA	1:5000	5% skim milk 0.05% TBST, RT 1 hour
Anti-rabbit IgG	Sigma, St. Louis, MO, USA	1:5000	5% skim milk 0.05% TBST, RT 1 hour

TBST, Tris buffered saline with Tween 20; RT, room temperature

Supplementary Table 4. Sequence of primers for quantitative real time RT-PCR

Gene	RefSeq ID	Forward primer	Reverse primer	Amplicon (bp)	Reference
PINK1	NM_032409.2	TTCCCCTTGGCCATCAAGA	ACCAGCTCCTGGCTCATTGT	86	1
Nix	NM_004331	TTGGATGCACAACATGAATCAGG	TCTTCTGACTGAGAGCTATGGTC	140	2
β-actin	AB004047	GTCCTCTCCCAAGTCCACAC	GGGAGACCAAAAGCCTTCAT	188	

[1]Seibler, P., *et al.* Mitochondrial Parkin recruitment is impaired in neurons derived from mutant PINK1 induced pluripotent stem cells. *J Neurosci* **31,** 5970-5976 (2011).

[2]PrimerBank, ID# 47078259c2
